# Steroid-Based Therapy and Risk of Infectious Complications

**DOI:** 10.1371/journal.pmed.1002025

**Published:** 2016-05-24

**Authors:** Lionel Rostaing, Paolo Malvezzi

**Affiliations:** 1 Clinique Universitaire de Néphrologie, Unité de Transplantation Rénale, CHU Grenoble, La Tronche, France; 2 Université Toulouse III Paul Sabatier, Toulouse, France; 3 INSERM U563, IFR–BMT, CHU Purpan, Toulouse, France

## Abstract

Lionel Rostaing and Paolo Malvezzi discuss the clinical implications of Laurence Fardet and colleagues' accompanying research study on infectious complications in patients receiving steroid treatment.

Steroid-based therapy is widely used in the treatment of different diseases, across many health care settings. In a study based on the National Health and Nutrition Examination Survey (NHANES), conducted between 1999 and 2005, the prevalence of long-duration glucocorticoid use in the United States general population was 1.2% (95% CI 1.1–1.4) [1]. In a study of the United Kingdom general population, over 20 years, the long-term use of glucocorticoids (>3 months) was 0.75% (95% CI 0.74–0.75) [2]. Over this time period, long-term corticosteroid prescriptions for rheumatoid arthritis, polymyalgia rheumatica/giant cell arteritis, ulcerative colitis, asthma, chronic obstructive pulmonary disease (COPD), and Crohn’s disease were evident. In addition, among patients with a solid-organ transplant, who number more than 1 million worldwide, at least 50% take 5–10 mg of glucocorticoids per day over the long term. With this in mind, it is of utmost importance that patients are managed in the best way with regards to side effects, particularly infectious complications.

What evidence is available about adverse events associated with steroid treatment? In 1958, Kass and Finland first reported on corticosteroid use and infections [3]. More recently, in a meta-analysis that included 28 studies (2,382 patients), the overall risk of adverse events was 150 per 100 patient-years (95% CI 132–169). Psychological and behavioral adverse events (e.g., minor mood disturbances) were reported most frequently, followed by gastrointestinal events (e.g., dyspepsia and dysphagia). Infectious complications included bacterial infections; reactivation of tuberculosis or toxoplasmosis; and viral infections, including herpes virus, varicella zoster virus, and reactivation of viral hepatitis [4].

In an analysis of pooled data from 71 randomized controlled trials that compared treatment with steroids versus no steroids, the overall rate of infectious complications was greater for those receiving steroids, i.e., the relative risk was increased by 60% (95% CI 30%–90%). However, infectious complications were not increased in those receiving a daily dose of <10 mg or a cumulative dose of <700 mg of prednisone [5]. More recently, a study examined the risk of nonserious infection in older patients with rheumatoid arthritis (*n* = 16,207) receiving chronic systemic glucocorticoids: the overall incidence of a nonserious infectious complication was 47.5 per 100 person-years, with an adjusted risk ratio increased by 20% (95% CI 15%–25%) in patients given glucocorticoids; in addition, a dose–response effect was evident [6].

In this issue of *PLOS Medicine*, Fardet and colleagues report on a study that examined common infections in UK patients prescribed systemic glucocorticoids in primary care [7]. Anonymized electronic medical data from patients were retrieved from THIN (The Health Improvement Network). In the UK, ~98% of the population is registered with a general practitioner. Participating general practitioners systematically and prospectively enter and retrieve clinical information on patients in THIN, including demographic data, diagnoses, and prescriptions: thus, this database provides a longitudinal medical record for each patient. Fardet and colleagues’ study used data entered between January 2000 and December 2012 for more than 275,000 patients prescribed oral glucocorticoids, along with patients not exposed to glucocorticoids.

The authors selected data on all synthetic glucocorticoids prescribed orally and recorded the start of treatment (date of the first prescription) and the end of treatment (date of the last prescription). In addition, the number of prescribed pills was available. In order to assess the effect of glucocorticosteroids on infection, they retrieved data on bacterial (i.e., sepsis, lower respiratory tract infection, and cutaneous cellulitis), viral (i.e., herpes zoster and varicella), fungal (i.e., mucocutaneous candidiasis and dermatophytosis), and parasitic (i.e., scabies) infections from THIN using the Read classification system. All patients with an infection recorded within the first 15 days of glucocorticoid initiation were excluded from the analyses, as the symptoms associated with the condition may have been the reason for prescribing rather than a consequence of glucocorticoid exposure.

Fardet and colleagues report that those patients prescribed glucocorticoids were more likely to have a history of diabetes, and were much more frequently exposed to other immunosuppressants than those not prescribed glucocorticoids. The underlying diseases of those prescribed glucocorticoids included asthma, COPD, cancer, polymyalgia/giant-cell arteritis, inflammatory bowel diseases, rheumatoid arthritis, and connective-tissue diseases. However, organ-transplant recipients were not included in the study.

The major finding of Fardet and colleagues’ study was that, compared to those with the same condition but not exposed to glucocorticoids, the hazard ratios for infection in the glucocorticoid-exposed population (57.8% women, median age 63 years) ranged from 2.01 (95% CI 1.83–2.19) for cutaneous cellulitis to 5.84 (95% CI 5.61–6.08) for lower respiratory-tract infection. In contrast, there was no difference in the incidence of scabies, dermatophytosis, or varicella between those who did and did not receive glucocorticoids. The increased relative risk was stable over the duration of exposure except for lower respiratory-tract infections and candidiasis, which were much more prevalent during the first weeks of exposure to glucocorticoids. In order to ensure that these variations in risk over time for lower respiratory-tract infections and candidiasis were not related to the greater prevalence of people with asthma and COPD in the study population, the authors ran a separate analysis of all patients but excluded those suffering from asthma or COPD. They found that the relative risks over time for developing respiratory-tract infections or candidiasis were similar to those of the overall population.

Fardet and colleagues also report that cancer patients, compared with other patients exposed to glucocorticoids, had the highest hazard ratios for septicemia (11.15, 95% CI 5.78–21.53) and local candidiasis (2.07, 95% CI 1.82–2.35). However, because data on chemotherapy for cancer patients were not included in the THIN database, it is difficult to know whether there was a link between treatment for cancer, prescription of oral glucocorticoids, and infection. In addition, overall, the authors found that the risk of infection was significantly increased in those with lower plasma-albumin concentrations. Because the data were anonymized, one could suggest that those patients with cancer were more likely to have hypoalbuminemia.

The study by Fardet and coworkers has some limitations—for example, physicians could have been more likely to report infections that occurred in glucocorticoid-treated patients than others, there were no data on adherence to glucocorticoids and no data on concomitant chemotherapy or targeted therapies for patients with rheumatoid arthritis, possible confounding factors were not identified, and solid-organ transplant patients were excluded. Yet, these findings still have major implications for general practitioners. Patients prescribed oral glucocorticoids are at high risk of developing a lower respiratory-tract infection: this has huge implications for patients with asthma or COPD. This implies the need for closer follow-up while on therapy as well as appropriate vaccination (i.e., against influenza or pneumococcus). Secondly, when prescribing oral glucocorticoids, the physician should carefully examine skin and mucosa in order to track mycosis (foot) or candidiasis (mouth). Finally, patients with cancer or diabetes, and those with hypoalbuminemia, have a much greater risk of infection when prescribed oral glucocorticoids and should, therefore, be informed of this and carefully monitored by their practitioner.

## Abbreviations

COPD: chronic obstructive pulmonary disease
NHANES: National Health and Nutrition Examination Survey
THIN: The Health Improvement Network
